# Evaluation of the Kushida Method of short-term earthquake prediction

**Published:** 2004-03-01

**Authors:** Seiya Uyeda, Atsumi Kumamoto

**Affiliations:** Earthquake Prediction Research Center, Tokai University, 3-20-1, Orido, Shimizu, Shizuoka 424-8610

**Keywords:** Earthquake prediction, VHF FM radio waves, alarm rate, success rate

## Abstract

Kushida and Kushida found that FM radio waves from stations at distances over-the-horizon are received before earthquakes. Based on this finding, since the mid-1990’s, the Kushidas have been practicing “Earthquake Precursor Detection Experiment”. The performance of the Kushida method during 2000–2003 has been evaluated by checking their predictions against the actual seismicity. During the period, there were 92 Kushida predictions mentioning the possibility of M ≥ 5.5 event, whereas there were 49 M ≥ 5.5 earthquakes in the Japanese region. If the criteria for successful prediction are set as: the errors in date is less than one day, epicentral position is roughly within specified area, and error in M is less than 0.5, the success rate was 20% and the alarm rate was 12%. If we relax the criteria to: the errors in dates within 10 days, epicenter within additional 100 km of specified area and the magnitude error less than 1.0, the success rate was 40% and the alarm rate was 27%. These rates may look insufficient for a practical prediction method. Considering, however, the fact that no other short-term prediction has ever been made in Japan so far it is a significant achievement. Moreover, it was found that in almost all failed predictions, meaningful signals were detected although the interpretations were incorrect. This indicates that the method is promising provided further investigation is carried out. The same evaluation at the M ≥ 6.0 level showed that the general performance was similar to the M ≥ 5.5 level, except that both success rate and alarm rate were lower at the M ≥ 6.0 level. If this unexpected finding is real, it might be inherent to the methodology using scattering of short-wave length radio waves as suggested by M. Hayakawa and may contain important information in understanding the earthquake physics and LAI-coupling. The results of the present study indicate strongly that the earthquake prediction research using anomalous transmission of VHF FM radio waves should be enhanced in parallel with complementary research in other frequency ranges.

## Introduction

Kushida and Kushida1) were monitoring VHF electromagnetic waves (~80 MHz) at Yatsugatake South Base Observatory, central Japan, for meteor observation. In the mid-1990’s, they unexpectedly received anomalous signals of FM radio waves from over-the-horizon stations before earthquakes. They interpreted this peculiar phenomenon as caused by some kind of scattering of FM radio waves which travel over the focal area of imminent earthquake and started an “Earthquake Precursor Detection Experiment”. Pilipenko *et al*.2) speculated its physical mechanism using existing notions of the “lithosphere-atmosphere-ionosphere coupling (LAI-coupling)”3): if some preseismic earth activity in a seismically active region generates gravity waves in the atmosphere, disturbances of electron density in the lower ionosphere may be formed with the result of scattering of VHF waves.

**Fig. 1:**
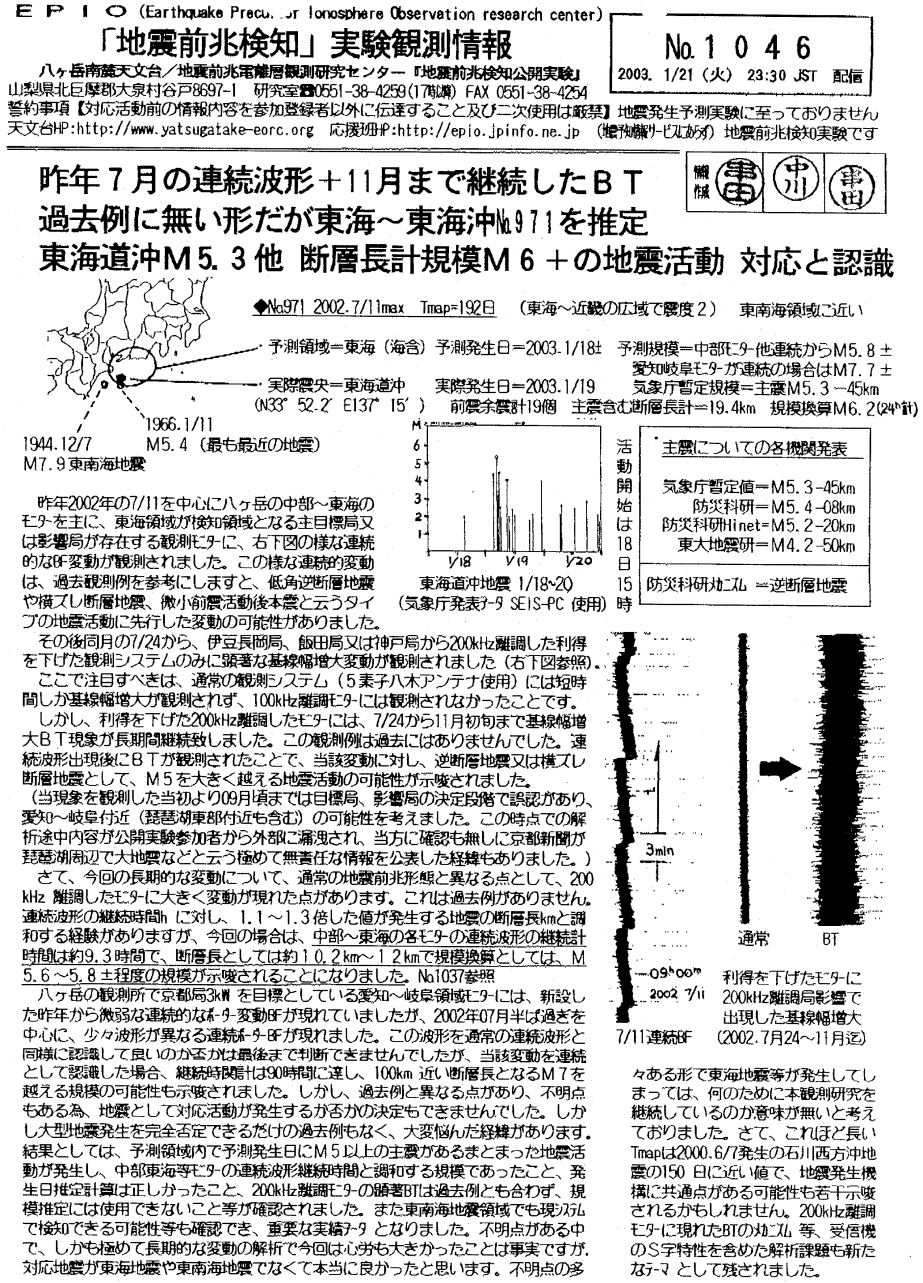
Kushida’s “Observation Information” News Letter No. 1046, issued on January 21, 2003, reporting the inferring process of the occurrence of January 19, 2003 M5.6 Off Tokai earthquake, mentioned in text. (With permission of Y. Kushida) The headline says: “Prediction No.971 (Tokai ~ Off Tokai event was estimated from the unprecedented continuous wavy signal of last July + BT (baseline thickness) anomalies continued to November. (These signals are shown in the figure in the right-hand column.) We recognize that the activity (M5.3 Off Tokai and the total fault-length magnitude6+) corresponds to the prediction.” M5.3 was JMA’s preliminary magnitude, which later was revised to M5.6. According to the Kushidas, the baseline on strip chart record thickens before some earthquakes. Also according to them, the sum of fault-lengths estimated for multiple quakes is supposed to represent the magnitude of the whole activity. See ref. 1) for details. Oval is the predicted epicentral area and black dot is real epicenter. Two dots with broken lines are the last earthquakes (1944 M7.9 and 1966 M5.4) in the area.

From numerous experiences, the Kushidas have derived some empirical rules1) for estimating the time, T, location, R, and magnitude, M, of impending earthquakes and have been practicing their “Earthquake Precursor Detection Experiment” for the last eight years. The Kushidas are now running three additional observing stations; i.e., in Hokkaido, Akita and in Shikoku aiming at numerous FM radio stations over the country. They would fax, 2–3 times a week, an “Observation Information (hereafter News Letter for simplicity)” describing up-to-date information on predictions and their results to the members of their supporting group. An example of a News Letter is shown in Fig. 1. News Letters are written in Japanese, but the example presented here may give some idea on how they are like. The content of this particular example will be mentioned later in this section. They limit the recipients of News Letters to the group members to avoid undue public unrest.

In the summer of 2003, however, they dared to publicize one of their predictions through media because they felt it obligatory to do so as they judged an M > 7 earthquake, that might devastate the metropolitan Tokyo area, was imminent. What actually happened was an M5.6 event which shook Tokyo area with JMA seismic intensity 4. Although this alarm was a failure because of the large magnitude error, it raised certain interests both in scientific and general communities. We, having long been paying attention to the Kushida method since its inception, believe that the method is now worth thorough cross-checking. Actually, such cross-check experiments have recently been started at several universities, including Hokkaido,4) Chiba,5) Tokyo Gakugei,6) Tele-Communications7) and Tokai universities.

At this time, it seems also important to evaluate the performance of the method objectively. Since their information is not open to non-members, objective checking has not been made since Yoshino *et al*., 1999 8) who examined the information up to December 1997 and stated that the method was promising. Here we report the up-dated results of our checking of the Kushida predictions.

## Method of checking

We evaluated the performance of the Kushida method by scrutinizing all of their 560 New Letters issued during the period 2000–2003. Their predictions were compared against the actual seismic activity tabulated in the catalogue of earthquakes published by the Japan Meteorological Agency (JMA).

Performance of earthquake prediction may be evaluated by two parameters. One is the success rate *S* and the other is the alarm rate *A* defined by,

$$\begin{array}{l} S=\frac{\text{number of successful predictions}}{\text{total number of predictions}},\\ A=\frac{\text{number of successfully predicted earthquakes}}{\text{total number of earthquakes}}. \end{array}$$

First, to evaluate these parameters, it is necessary to define the population of predictions and earthquakes to deal with. It would be reasonable to focus our attention to large earthquakes and disregard small ones. For evaluating *S*, only predictions which mentioned the occurrence of M ≥ 5.5 earthquakes in their statement were selected from Kushida News Letters. (They will be abbreviated as “M ≥ 5.5 predictions”.) Then, we examined if they were successful by checking with the JMA catalogue. In doing so, we looked at M ≥ 4.5 earthquakes only, disregarding M < 4.5 earthquakes.

Here, a care was taken in counting the number of predictions. Kushida’s signals often show time evolutions, which are sometimes complicated. In such a case, statements in consecutive News Letters also change. As long as the changes were made before the predicted dates of the earthquake in question, they were lumped together and considered as one prediction. For example, prediction of the January 19, 2003 M5.6(JMA final) earthquake off Tokai coast started several months before, i. e., July 11, 2002 (News Letter #971). Until the last prediction was made on December 28, 2002 (News Letter #1037), almost 20 statements, some of which were of different contents, were issued. We count them as one prediction. News Letter #1046 of January 21, 2003 shown in Fig. 1 summarizes the whole story on the predictions related to this earthquake. Actually, the area of that earthquake has been quiet for about 40 years since 1966, so that the epicenter estimation made three weeks before the occurrence may be considered a significant feat. On the other hand, when a change was made after the predicted date of the earthquake occurrence, preceding related statements were counted as one false alarm.

For the evaluation of *A*, only M ≥ 5.5 earthquakes in Japan and its immediate environs were selected from the JMA catalogue and examined which of them were successfully predicted. Earthquakes deeper than 100 km, aftershocks of major earthquakes, earthquakes that occurred in the area outside the “detectability area”, and earthquakes that occurred while the system was out of order were excluded from the population. Non-detectability areas are empirically inferred. Faraway regions are obviously non-detectability areas, but some areas such as parts of the Pacific side of the Tohoku region were also designated non-detectable area. According to Kushida, the emitter-receiver-epicenter geometry, focal depth and water depth at sea may be key factors in dictating the detectability and non-detectability. However, geographic distribution of detectable and non-detectable areas has not been clearly delineated yet.

Second, it is necessary to set the criteria for success-failure decision. Successful predictions must specify the time, T, location, R, and magnitude, M, within some permissible error ranges, ΔT, ΔR, and ΔM. We adopted two levels of criteria as follows:

**Table:** 

Criteria A:	ΔT ≤ ± 1 day of predicted time window, which is generally a few days, sometimes longer.
	ΔR = Roughly within predicted circular or oval area. By roughly, it is meant that ~20 km out of the area is allowed. The size of predicted area varies but are usually a few to several tens of km in radius, sometimes larger.
	ΔM ≤ ± 0.5 of predicted magnitude. Prediction is sometimes given in an M window, such as 4.5–5.5. In such a case, permissible range of M is 4.0–6.0, but it will in fact be 4.5–6.0, because we disregard M < 4.5 earthquakes.
Criteria B:	ΔT ≤ ± 10 days of predicted time window.
	ΔR ≤ within 100 km of predicted area cited above.
	ΔM ≤ ± 1 of predicted M window.

It must be noted that, according to the above evaluation system, the lower limit of the magnitude of earthquakes for the success rate count can be as low as 4.5, whereas that for the alarm rate is 5.5, because M < 5.5 earthquakes were not the objects of alarm rate evaluation.

A prediction is scored successful only when all three conditions are satisfied in each criteria. Criteria A are practically the same as the Kushidas themselves impose on their predictions. Since they are extremely stringent, we set the more relaxed alternative criteria B, which are still reasonably high.

## Results

### Success rate

**Fig. 2:**
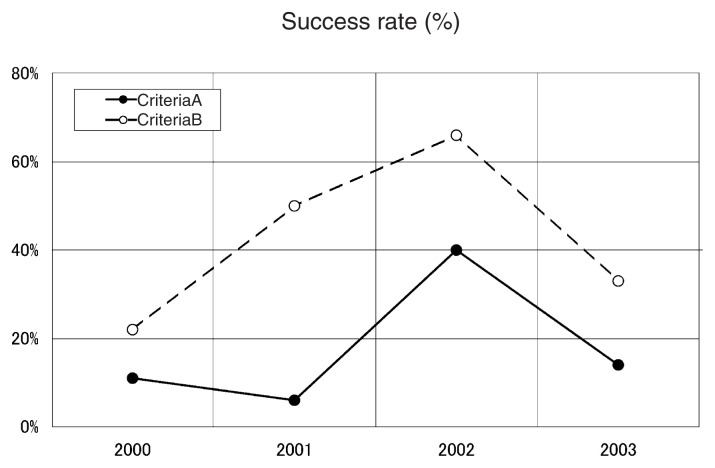
Success rate of “M ≥ 5.5 predictions” for the period 2000–2003.

**Fig. 3:**
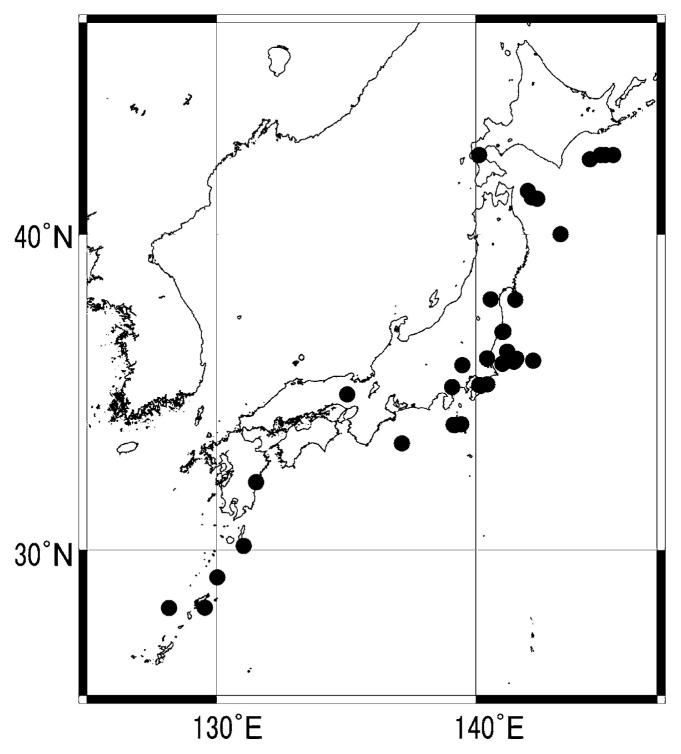
Geographic distribution of earthquakes predicted successfully under criteria B by “M ≥ 5.5 predictions”.

Table I shows the total number of predictions which mentioned the occurrence of M ≥ 5.5 earthquakes, i.e. “M ≥ 5.5 predictions”, the number of successful such predictions and their ratios, namely the success rate (Fig. 2) for each year and for the four years. As expected, there were more successes under criteria B than A. For the four year period, the rates are 20% under criteria A and 40% under criteria B. Fig. 3 shows the distribution of earthquakes successfully predicted under criteria B by “M ≥ 5.5 predictions”. It must be reminded that the earthquakes in Fig. 3 are not always greater than 5.5 in magnitude owing to the adopted criteria: an M4.5 earthquake can be one of them if an “M5.5 prediction” met the criteria B.

The rate varied year to year (Fig. 2): the years 2001 and 2000 gave the poorest score for criteria A and B, respectively. These variations may reflect the fluctuations for the small population. But a closer look of the News Letters indicated that many predictions in the year 2000 counted as individual false alarms were, in hindsight, concerned with some common signals. If those related to common signals are lumped together, the total number of predictions becomes 37 instead of 45, so that the rates may be raised to 14% (criteria A) and 27% (criteria B). Why this happened mainly in the year 2000 is not clear.

In the summer of 2000, there was a large swarm activity with 11 M ≥ 5.5 events in the Izu island region.9) Seven “M ≥ 5.5 predictions” were issued for this swarm activity. Among them, five (News Letters 714-2, 715-1, 717-1, 717-2, and 724-1) were successful even under criteria A, while two were false (719-1 and 721-2) even under criteria B. Although the annual success rate *S* was low in 2000, the success rate for this swarm was 71%. This high rate, however, could be by chance because of the high swarm activity (see below).

### Alarm rate

**Fig. 4:**
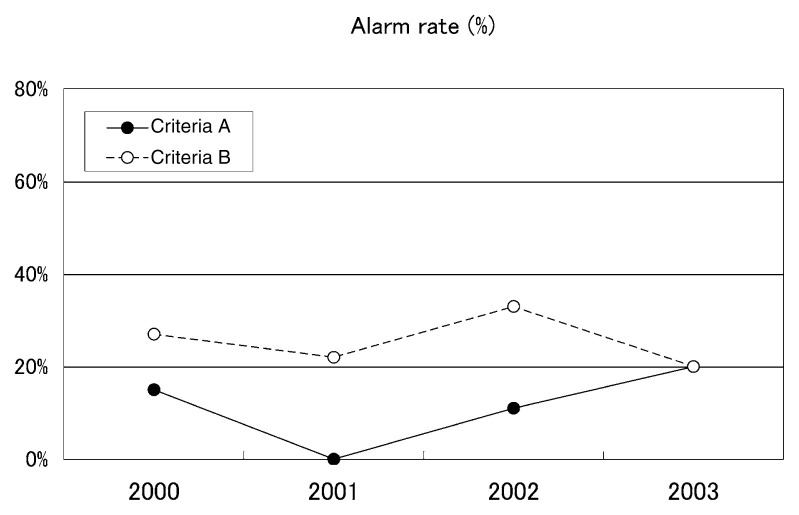
Alarm rate of M ≥ 5.5 earthquakes for the period 2000–2003.

**Fig. 5:**
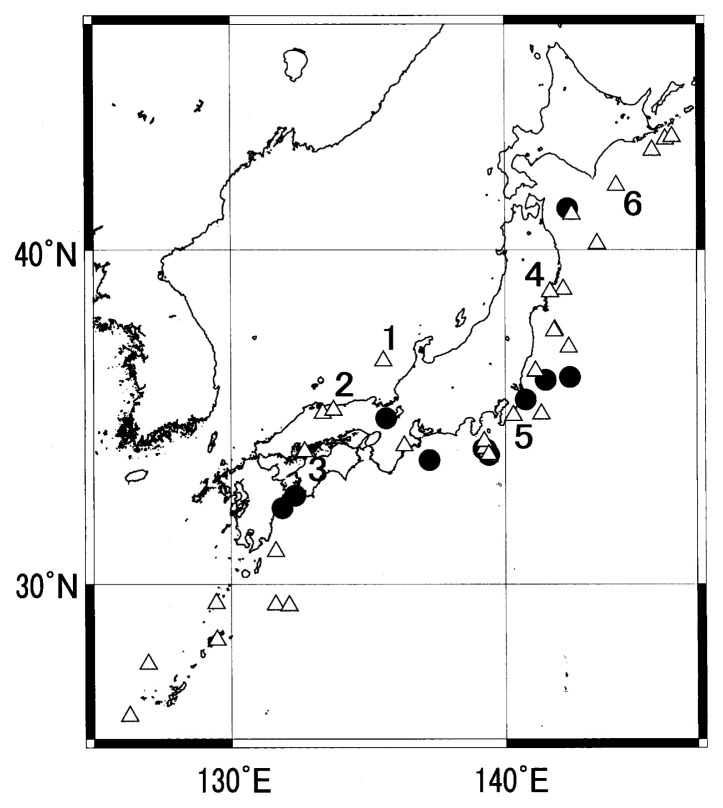
Successfully predicted M ≥ 5.5 earthquakes (full circles) and un-predicted or unsuccessfully predicted M ≥ 5.5 earthquakes (triangles). Numbers attached to some triangles indicate the earthquakes described in the section on false alarms.

Table II shows the total number of earthquakes (earthquakes deeper than 100 km, in non-detectability regions and aftershocks were excluded) in the Japanese region, the number of successfully predicted M ≥ 5.5 earthquakes and their ratios, namely the alarm rate (Fig. 4) for each year and for the four years. Again as expected, the score is better for criteria B than A. The alarm rate also shows some variation for different years. For the four year period, the rates are 12% under criteria A and 27% under criteria B. Geographic distribution of successfully predicted and un-predicted M ≥ 5.5 earthquakes (both under criteria B) are shown in Fig. 5. It must be noted that Fig. 3 and Fig. 5 look alike but they are different because Fig. 5 contains only M ≥ 5.5 earthquakes. Un-predicted earthquakes may well be called unsuccessfully predicted because the predictions were made except the case 4 in the figure which will be mentioned later. These predictions, however, simply did not meet the criteria. Though not clear, it may be observed that the Kushida method during the period 2000–2003 had difficulty in successfully predicting earthquakes in the Pacific side of Tohoku, and Chugoku and Nansei Shoto regions.

## On false alarms

There were large earthquakes which were not successfully predicted. The case history of some notable failures is as follows: It seems that some relevant signals were recognized but the interpretations were incorrect.

The June 7, 2000 M6.2 Off the Western Ishikawa Prefecture earthquake (No. 1 in Fig. 5).

Starting from December 8, 1999 (News Letter #613), more than 10 News Letters carried its prediction. Of the two candidate positions, Kushida finally (June 4, 2000, News Letter #694) discarded the Off the Western Ishikawa Prefecture (the right one) and adopted the inland candidate (Nagano Prefecture) wrongly because the signals looked similar with those of a previous inland earthquake. The results were ΔT = 0, ΔR = 200 km, and ΔM = 0.2. The large ΔR disqualified this prediction.

The October 6, 2000 M7.3 Western Tottori earthquake (No. 2 in Fig. 5).

This was a major inland earthquake during the period. Starting from August 13, 2000 (News Letter #724), many News Letters carried its predictions with candidate epicenter at varied areas in Japan Sea, Philippine Sea and Kanto area. On October 6 immediately before the earthquake (News Letter #751), the signals were wrongly attributed to volcanic activity in the Izu region.

The March 24, 2001 M6.7 Geiyo earthquake (No. 3 in Fig. 5).

This was also a major inland earthquake for the period. From February 1, 2001 (News Letter #796), more than ten News Letters dealt with this prediction with possible epicenter in Wakasa Bay, and western Chugoku (which was right). But on March 4 (News Letter #811), the signals were wrongly attributed to volcanic activity in the Izu region.

The July 26, 2003 M6.4 Northern Miyagi Prefecture earthquake (No. 4 in Fig. 5).

It shook Sendai area with intensity 6 in JMA scale, but no prediction was made because the epicenter was out of detectability region according to News Letter #1132 (July 27, 2003).

The September 20, 2003 M5.6 Southern Chiba Prefecture earthquake (No. 5 in Fig. 5).

Prediction started on February 15, 2003 (News Letter #1057), and a number of predictions followed inferring possible epicenter in Tokai region and Off the Kanto areas. Public announcement (in a popular weekly magazine), mentioned in the Introduction, was made on September 8, alarming the possibility of devastating M7.2 earthquake on around September 17, 2003. The results were ΔT = 3 days, ΔR ~100 km, and ΔM =1.6. The large ΔM disqualified this prediction.

The September 26, 2003 M8.0 Off Tokachi earthquake (No. 6 in Fig. 5).

This was the largest event in the period. On August 3, 2003 (News Letter #1136), two locations were suggested for an earthquake; one in Japan Sea and the other Off Tokachi. On September 25 (one day before the earthquake), a brief prediction was made as “~M5–6 earthquake is expected at Off Tokachi in a few months”. Thus, it was a false prediction on account of both M and time error. Kushida remarks he was too busy with the September 20 earthquake to pay due attention to this earthquake.

## Discussion

### Success by chance?

**Fig. 6:**
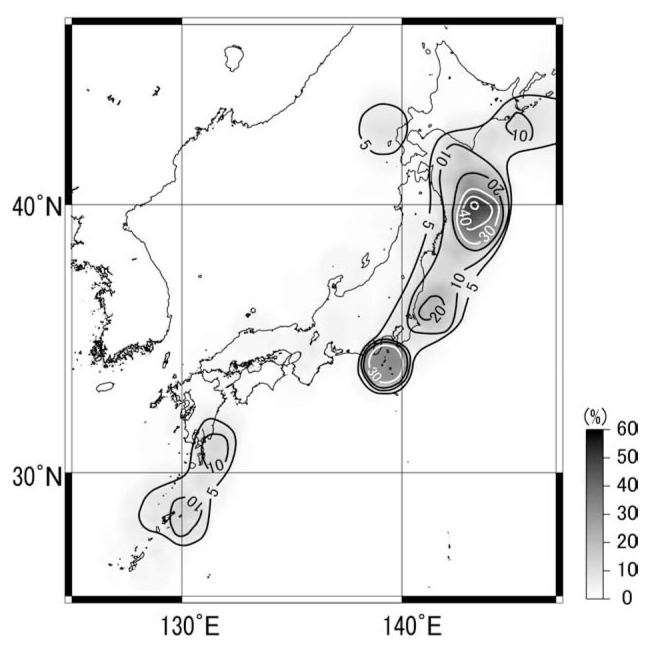
Distribution of chancy success of hitting an M ≥ 5.5 earthquake by a randomly issued M ≥ 5.5 prediction with time and epicentral window of 20 days and a circle with radius 100 km. The contours were inferred from the JMA catalogue of earthquakes for 1991–2000.

As shown in Figs. 3 and 5, many successful predictions were made in Japan. But the seismicity of the region is high. There may be a possibility of chancy success. Fig. 6 shows the contour map of the roughly estimated rate of chancy success in % when a prediction is made for a M ≥ 5.5 earthquake with twenty days and 100 km of time and space windows. The contour map is based on simply counting the number of M ≥ 5.5 earthquakes in the JMA catalogue for 1991–2000 for circular areas with radius 100 km and multiplying the number by 20days/3650days. The chance seems as high as 40% off the northeastern Honshu, where only few predictions were made because that region tends to be “undetectable” according to Kushida. In the area south of Tokyo, Kushida predictions scored 70% of success for the swarm of the summer of the year 2000. Since the chance of 30% shown in Fig. 6 in this region is the average of ten years, it could have been much higher at the time of the swarm. Therefore, the 70% of success could well have been by chance, even though the time windows in most of the Izu swarm predictions were only a couple of days. In most of the rest of the Japanese region, however, the chance of success of random prediction is lower than, say 5%. In such areas as western Japan, the success of Kushida predictions were definitely higher than chance. Furthermore, the failure history of predictions for the earthquakes Nos. 1, 2 and 3 (Fig. 5) depicted earlier indicates that the experiences will probably make the prediction successful next time. If that happens, the method will be more credible because the area is of almost zero % of chancy success.

### Lower success for larger earthquakes?

Another matter of potential importance of the Kushida method is that it seems to fail for larger earthquakes. Some case histories were depicted in the previous section. Exactly the same evaluation as done at the M ≥ 5.5 level has been made at the M ≥ 6.0. Table III is the summary comparison of the estimated rates for M ≥ 5.5 and M ≥ 6.0 evaluations. In each table, the upper line is a copy from Table I and II. Table III shows that both success and alarm rates under either criteria A or B are lower for the M ≥ 6.0 level evaluation than for the M ≥ 5.5 level. Although there is a possibility that sample numbers are not large enough to draw general conclusion, this result contradicts usual expectation: namely if earthquake precursors exist, it would be larger for larger earthquakes and the prediction would be easier. Actually, the alarm rate of the VAN predictions was demonstrated to increase dramatically with earthquake magnitude.10) In the Kushida method, too, signals are actually lager for larger earthquakes and their magnitude estimate is based on this fact.1) Why is the score lower for larger earthquakes? Two potential reasons for this apparent enigma are conceivable. One is their experience that volcanic activity produces strong VHF FM radio wave anomaly. Some convincing evidence has been reported in their News Letters for the activities at volcanoes, including Sakurajima (1999), Usu (2000), and Miyake (2000). From these experiences, Kushida sometimes interpreted strong signals as volcanic precursors (see preceding section). Volcanic activity may be surface eruptive activity or earthquake activity of small magnitudes under volcanoes. Since these activities are hardly quantified at this stage, evaluation of their prediction on volcanic activity has not been made. This will be an important next subject. The other reason, which may be of more direct relevance to the physics involved in earthquake generation, is that the time and space scales of the appearance of scatterers of the VHF FM radio waves for large earthquakes may also be large, and the mode of appearance may be complicated in both time and space. If such was the case, predictions tend to become wayward and success would become more difficult. M. Hayakawa (private communication) suggests that time-space heterogeneous appearance of scatterers may be due to the short wave length of VHF radio waves and joint use of similar approach with VLF waves with much longer wave length would not only improve the prediction technology, but would make significant contribution to understand the physics of LAI-coupling.2)

## Conclusion

The performance of the Kushida method during 2000–2003 has been evaluated at the M ≥ 5.5 and M ≥ 6.0 levels. During the period, there were 92 “M ≥ 5.5” and 44 “M ≥ 6.0” predictions, whereas there were 49 M ≥ 5.5 and 21 M ≥ 6.0 earthquakes. Under the criteria for successful prediction that ΔT less than 1 day, ΔR within specified area, and ΔM less than 0.5 Richter units, the success rate was 20% and 18% and the alarm rate was 12% and 5% for the two levels. If we allow the errors to 10 days, within additional 100 km of specified area and 1.0 Richter units, the success rates were 40% and 38% and the alarm rates were 27% and 19% respectively. These rates were much higher than chancy success. Moreover, it was found that in most of false predictions, meaningful signals were detected although their interpretation led unsuccessful results, indicating the possibility that their performance may well be improved by accumulating further experience. As mentioned above, it was also found that the performance of the Kushida method may be lower for earthquakes larger than 6 in magnitude. If this is a real fact, it may provide important information on the physics involved. Although the Kushida method is still far from perfect, it seems that the over-the-horizon transmission of VHF FM radio waves before earthquakes may well be real and serious efforts should be devoted to improve the methodology and clarify the underlying physics.

## Figures and Tables

**Table I:** Annual values of success rate *S* of “M ≥ 5.5 predictions”

Year	M ≥ 5.5 predictions	Successful predictions		Success rate *S* (%)	
	
		Criteria A	Criteria B	(A)	(B)
2000	45	5	10	11%	22%
2001	12	1	6	8%	50%
2002	26	11	18	42%	69%
2003	9	1	3	11%	33%

2000–2003	92	18	37	20%	40%

Bottom line shows the values of four years. For definition of “M ≥ 5.5 prediction” and criteria A and B, see text.

**Table II:** Annual values of alarm rate *A* for M ≥ 5.5 earthquakes

Year	M ≥ 5.5 earthquakes	Predicted earthquakes		Alarm rate *A* (%)	
	
		Criteria A	Criteria B	(A)	(B)
2000	26	4	7	15%	27%
2001	9	0	2	0%	22%
2002	9	1	3	11%	33%
2003	5	1	1	20%	20%

2000–2003	49	6	13	12%	27%

Bottom line shows the value of four years. For criteria A and B, see text.

**Table III:** Comparison of four year values of success rate *S* and alarm rate *A* evaluated at M ≥ 5.5 level and M ≥ 6.0 level. Rates were lower for larger earthquakes.

Success rate *S* (%)					

Number of predictions		Successful predictions		Success rate(%)	
	
		Criteria A	Criteria B	(A)	(B)
M ≥ 5.5	92	18	37	20%	40%
M ≥ 6.0	44	8	17	18%	38%

Alarm rate *A* (%)					

Number of earthquakes		Predicted earthquakes		Alarm rate (%)	
	
		Criteria A	Criteria B	(A)	(B)
M ≥ 5.5	49	6	13	12%	27%
M ≥ 6.0	21	1	4	5%	19%
